# Isocorydine Inhibits Cell Proliferation in Hepatocellular Carcinoma Cell Lines by Inducing G2/M Cell Cycle Arrest and Apoptosis

**DOI:** 10.1371/journal.pone.0036808

**Published:** 2012-05-18

**Authors:** Hefen Sun, Helei Hou, Ping Lu, Lixing Zhang, Fangyu Zhao, Chao Ge, Tingpu Wang, Ming Yao, Jinjun Li

**Affiliations:** 1 State Key Laboratory of Oncogenes & Related Genes, Shanghai Cancer Institute, Renji Hospital, Jiaotong University School of Medicine, Shanghai, China; 2 Experimental Pathological Laboratory, Shanghai Cancer Institute, Renji Hospital, Jiaotong University School of Medicine, Shanghai, China; 3 College of Life Sciences and Chemistry, Tianshui Normal University, Tianshui, China; The University of Hong Kong, China

## Abstract

The treatment of human hepatocellular carcinoma (HCC) cell lines with (+)-isocorydine, which was isolated and purified from *Papaveraceae sp.* plants, resulted in a growth inhibitory effect caused by the induction of G2/M phase cell cycle arrest and apoptosis. We report that isocorydine induces G2/M phase arrest by increasing cyclin B1 and p-CDK1 expression levels, which was caused by decreasing the expression and inhibiting the activation of Cdc25C. The phosphorylation levels of Chk1 and Chk2 were increased after ICD treatment. Furthermore, G2/M arrest induced by ICD can be disrupted by Chk1 siRNA but not by Chk2 siRNA. In addition, isocorydine treatment led to a decrease in the percentage of CD133^+^ PLC/PRF/5 cells. Interestingly, isocorydine treatment dramatically decreased the tumorigenicity of SMMC-7721 and Huh7 cells. These findings indicate that isocorydine might be a potential therapeutic drug for the chemotherapeutic treatment of HCC.

## Introduction

Hepatocellular carcinoma (HCC) is the fifth most common cancer in the world [1]. Although many anti-cancer drugs have been used in the routine clinical treatment of HCC patients and result in a reduction in tumor volume at early stages, recurrence, the development of multi-drug resistance, toxicity and side effects are unfortunately common in patients. Therefore, there is a pressing need for new therapeutic drugs with increased efficacy and decreased toxicity.

Cell cycle deregulation is a hallmark of tumor cells, and targeting the proteins that mediate critical cell cycle processes is an emerging strategy for the treatment of cancer [2]. The G2/M checkpoint is the most conspicuous target for many anticancer drugs [3], [4]. CDK1/cyclin B1 and CDK1/cyclin A complexes play a key role in promoting the G2/M phase transition. Many proteins are known to regulate the stepwise activation of CDK1, which controls the G2 to M transition. This process involves additional proteins, including Weel [5], Myt1 [6], Cdc25C [7] and others. The phosphatase activity of Cdc25C is inactivated by Chk1/Chk2, which are activated by ATM/ATR in response to DNA damage.

In the past few years, it has been demonstrated that extracts from several medicinal plants that are used in traditional medicine can inhibit tumor proliferation. These plants possess a wide spectrum of biological activities, including anti-bacterial and fungicidal properties [8]. Alkaloids from *Dicranostigma leptopodum (Maxim.) Fedde* (DLF) possess antipyretic activity and have been used in the clinical treatment of pulmonary tuberculosis. Whether these alkaloids also have anti-cancer effects against HCC is poorly understood. In this study, we demonstrate that components present in DLF extracts can inhibit the growth of HCC cells by inducing both G2/M cell cycle arrest and apoptosis. The major components present in DLF extracts include dicranostigmine, isocorydine, corydine, protopine and sinoacutine [9]. We found that L-(+)-isocorydine (ICD) could be isolated and purified from *Papaveraceae sp.* plants such as *Dactylicapnos scandens Hutchins* and *Dicranostigma leptopodum (Maxim.) Fedde*. In our pilot study, ICD was found to play a key role in the anti-proliferative effect observed in various HCC cell lines. The main goals of the present study were to demonstrate the anti-cancer effect of purified ICD and to delineate the underlying mechanisms of its anti-cancer properties. The chemical structure of ICD is shown in Figure S1.

## Results

### Dicranostigma Leptopodum (Maxim.) Fedde (DLF) Extract Suppresses the Proliferation of HCC Cell Lines through G2/M Arrest

**Figure 1:**
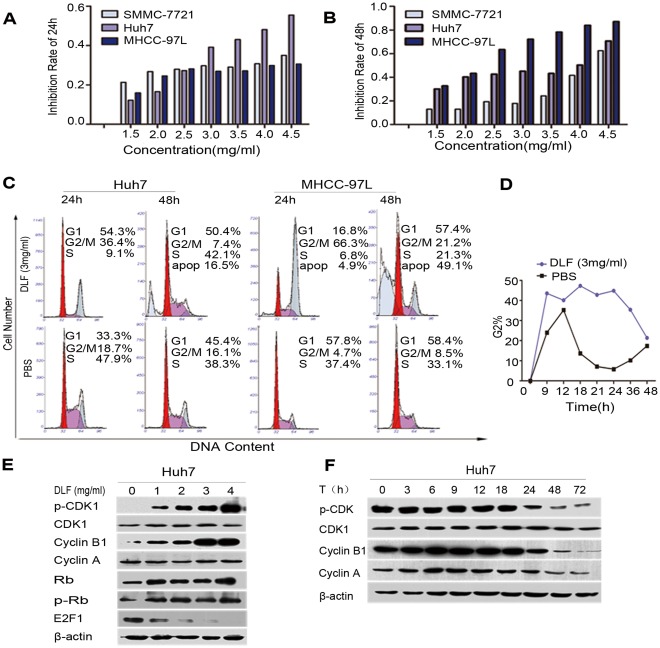
The effect of DLF on HCC cell growth and cell cycle-related protein expression *in vitro*. (A, B) Growth inhibition resulting from the treatment of HCC cells with DLF extracts for 24 h and 48 h. (C) The cell cycle distribution of Huh7 and MHCC-97L cells that were treated with 3 mg/ml DLF for 24 h or 48 h. (D) The percentage of Huh7 cells in G2 phase following treatment with DLF extracts for various times. (E, F) Western blot analysis of the expression of G2/M phase transition-related proteins after treatment with DLF extracts at various concentrations and for various times.

To investigate the suppressive growth effect of the DLF extracts, the HCC cell lines SMMC-7721, MHCC-97L and Huh7 were incubated with various concentrations of DLF extracts for 24 and 48 h. Cell proliferation was subsequently measured by the MTT assay. Our results show that the DLF extracts could inhibit the growth of all three HCC cell lines in a dose- and time-dependent manner (Figure 1A, B).

To further elucidate the inhibitory effects of the DLF extracts on HCC cell growth, the cell cycle distributions of the three cell lines were determined by flow cytometry. Following treatment with 1 mM thymidine for 24 h to synchronize cells at the G1/S border, the cells were incubated with 3 mg/ml of DLF extracts for various times. A dose-dependent G2/M arrest in the cell cycle was observed in Huh7 and MHCC-97L cells after treatment for 24 h with DLF extracts. An apoptotic, sub-G_0_ population of cells was also observed at the 48 h time point (Figure 1C). We observed that the accumulation of cells at the G2/M phase could persist for 48 h (Figure 1D).

We next examined the expression of the key molecules that promote the G2/M phase transition. Western blot analysis showed that treatment with the DLF extracts increased the expression of cyclin B1 and the inhibitory phosphorylation status of CDK1 (Tyr15) in a dose-dependent manner. Because an active CDK1/cyclin B1 complex is known to promote the transition between the G2 and M phases, the accumulation of p-CDK1 (Tyr-15) indicated the presence of an inactive complex. The classical model of the E2F/p-Rb complex suggests that it is the predominant complex that promotes cell cycle transition. Therefore, to study the effect of DLF extracts on the E2F1/p-Rb complex, Western blotting was used to analyze the expression of E2F1 and p-Rb. The results showed that the DLF extracts did not affect the phosphorylation status of Rb; however, they did decrease the expression level of E2F1 (Figure 1E). The p-CDK1 and cyclin B1 levels were maintained for 48 h (Figure 1F), thus validating the cycle analysis results shown in Figure 1D.

### ICD Inhibits Cell Proliferation and Induces Apoptosis

The DLF extracts is a mixture that contains more than five components [10]. Therefore, to determine which component plays the major role in suppressing cell growth, we obtained one of the main components, (+)-Isocorydine (ICD), and investigated its suppressive effects on tumor cells using the MTT assay.

**Figure 2:**
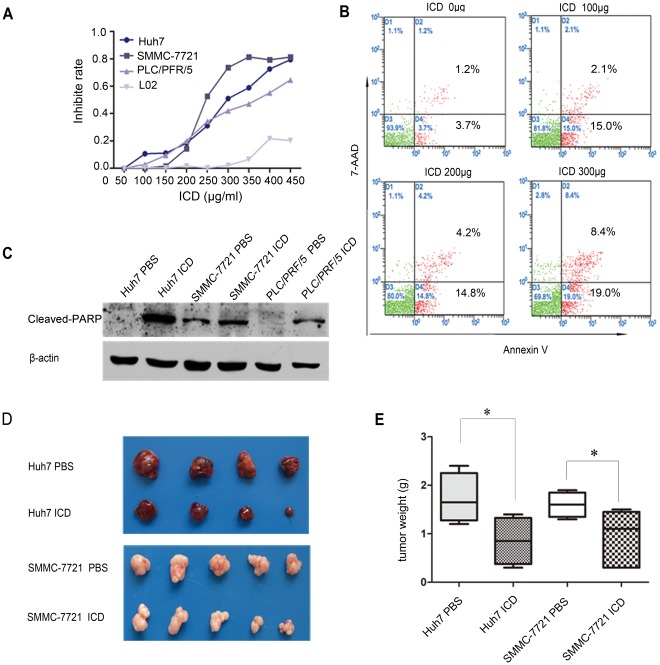
DLF extracts causes cell growth inhibition and apoptosis. (A) The growth inhibition rates of Huh7, SMMC-7721, PLC/PFR/5 and L-02 cells resulting from treatment with ICD for 48 h. (B) Following the treatment of Huh7 cells with 0, 100, 200 or 300 µg/ml ICD for 48 h, apoptotic cells were detected by Annexin V and 7-AAD double staining. (C) Western blot analysis of cleaved PARP in Huh7, SMMC-7721 and PLC/PRF/5 cells following ICD treatment at their respective IC_50_ values (250 µg/ml, 200 µg/ml, 250 µg/ml). (D) An amount of 1×10^6^ Huh7 or SMMC-7721 cells/mouse was subcutaneously injected into nude mice. After 2 weeks, 0.4 mg/ml of ICD or PBS was administered 5 times per week for 4 weeks. The resulting tumors were excised from the animals after treatment. (E) The tumor weights for the four groups of animals were compared, and statistical significance was determined using the Student’s *t*-test. Each point represents the mean ± SD.

ICD was found to inhibit the growth of Huh7, SMMC-7721 and PLC/PRF/5 cells in a dose-dependent manner when applied for 48 h (Figure 2A). To exclude the possibility that cell death was occurring due to drug toxicity, the effect of ICD on the immortalized human liver cell line L-02 was also investigated. L-02 cells were found to have a low sensitivity to ICD treatment and exhibited an inhibition rate that was less than 1% of that observed for each of the HCC cell lines at their IC_50_ values (200, 250 and 300 µg/ml for SMMC-7721, Huh7 and PLC/PRF/5, respectively). The AKT and Erk1/2 pathways are known to play crucial roles in the promotion of cell survival and the inhibition of apoptosis; however, ICD did not have any effect on the expression levels of p-AKT, p-S6 or p-Erk1/2 (Figure S2).

To confirm that ICD treatment induces apoptosis, Huh7 cell apoptosis was examined using the Annexin V/7-AAD double-staining method and flow cytometry analysis. Our results revealed that treatment with ICD at 100, 200 and 300 µg/ml for 48 h increased the percentage of apoptotic cells to 17.1%, 19.0% and 27.4%, respectively (Figure 2B). We also found that ICD could induce apoptosis in SMMC-7721 cells (data not shown). An upregulation of cleaved PARP was detected via Western blotting in all three HCC cell lines after a 48 h treatment, indicating that ICD may induce apoptosis through PARP cleavage (Figure 2C). We used real-time PCR to detect the messenger RNA levels of several apoptosis-related genes. We observed an upregulation in the expression of CYP27A1, TNFSRSF10 and TNFSRSF9 after ICD treatment (Figure S3).

Because ICD was able to inhibit cell proliferation *in vitro*, we next investigated the effects of ICD on HCC tumorigenicity *in vivo* using a mouse xenograft model. The body weights of the ICD-treated groups, which were inoculated with Huh7 or SMMC-7721 cells, were 23.1±2.6 g and 27.4±1.5 g, respectively. No significant difference in body weight was observed between the inoculated mice and the control mice, which had body weights of 24.6±0.5 g (Huh7; *p*>0.05) and 26.6±0.8 g (SMMC-7721; *p*>0.05), respectively. These results indicate that ICD does not induce toxicity in mice. The tumor weights of the xenografts for both ICD and control groups are shown in Figure 2D and E. Our results indicate that ICD significantly inhibits the tumorigenicity of Huh7 (*p*<0.05) and SMMC-7721 (*p*<0.05) in this model system.

### ICD Induces G2/M Cell Cycle Arrest by Decreasing the Activity of CDK1

**Figure 3:**
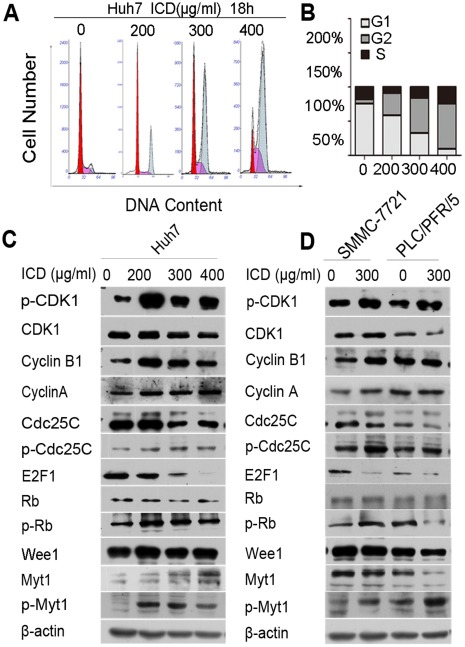
ICD treatment causes cells cycle arrest at the G2/M phase. (A) The cell cycle distribution of Huh7 cells that were treated with various doses of ICD for 18 h. (B) A statistical graph of the cell cycle distribution shown in (A). (C and D) Western blot analysis of G2/M transition-related proteins after ICD treatment of Huh7, SMMC-7721 and PLC/PRF/5 cells for 18 h.

To verify that ICD can cause cell cycle arrest, the Huh7 and SMMC-7721 cell cycles were analyzed following treatment with ICD at different concentrations and for different times. The results showed that ICD induced G2/M cell cycle arrest to the same extent as did the DLF extracts. In the Huh7 cell line, the percentages of cells in G2/M phase were 6.2%, 32.7%, 51.3% and 66.2% after an 18 h treatment with ICD concentrations of 0, 200, 300 and 400 µg/ml, respectively (Figure 3A, B). We also observed cell cycle arrest in SMMC-7721 and Huh7 cells after ICD treatments of different times and concentrations (summarized in Table 1, 2). In addition, the G2/M phase arrest was confirmed by Western blotting in Huh7 cells following ICD treatments of 0, 200, 300 and 400 µg/ml for 18 h (Figure 3C). ICD treatment led to an upregulation in the expression level of cyclin B1, but it did not affect the expression level of cyclin A. Furthermore, ICD treatment led to a decrease in CDK1 activity, as we observed an accumulation of Thr-14/Tyr-15-phosphorylated (inactive) CDK1. The Cdc25C protein activates the cyclin B1/CDK1 complex by dephosphorylating these inhibitory residues on CDK1. We found that ICD treatment resulted in an increase in the p-Cdc25C protein level and a decrease in the total level of Cdc25C. Therefore, these data indicate that ICD may inactivate Cdc25C through an inhibition of the CDK1/cyclin B1 complex, thereby inducing G2/M phase arrest. Phosphorylation of CDK1 at Tyr15 and Thr14 sites is known to be performed by the Wee1 and Myt1 protein kinases. We observed an upregulation in the expression level of p-Myt1 protein following ICD treatment in Huh7 cells; however, ICD did not affect the expression level of Wee1. Similar results were observed in SMMC-7721 and PLC/PRF/5 cells (Figure 3D).

**Table 1:** Cell cycle distribution of Huh7 HCC cells after ICD treatment with gradient concentrations.

Time	Cell cycle	ICD (µg/ml)			
		0	200	300	400
**9 h**	**G1**	31.588±2.183	12.850±0.372	3.488±2.232	6.064±0.087
	**S**	15.387±2.637	32.559±3.719	32.586±1.917	20.16±1.543
	**G2/M**	53.025±4.567	54.590±3.346	**63.925±0.413** *	**73.775±1.621** **
**12 h**	**G1**	62.842±0.780	17.716±7.334	15.582±1.849	11.190±5.711
	**S**	11.709±1.287	26.335±9.468	19.9±4.311	17.294±6.634
	**G2/M**	25.448±2.068	**55.948±2.205** **	**64.517±6.056** **	**71.515±10.022** **
**15 h**	**G1**	64.100±0.307	32.157±4.263	24.031±2.747	22.274±0.003
	**S**	9.520±2.065	14.450±5.976	14.195±1.169	12.541±0.996
	**G2/M**	26.379±1.759	**53.392±2.681** **	**61.773±3.917** **	**65.184±0.993** **
**18 h**	**G1**	76.032±4.581	45.302±2.051	33.035±0.882	12.417±4.656
	**S**	13.657±4.183	3.905±1.231	15.936±2.302	20.571±3.812
	**G2/M**	9.643±3.747	**50.792±0.843** **	**51.027±3.184** **	**67.011±1.949** **

Data are mean ± SD of three independent experiments.

* Significant differences (*p*<0.05),

** Significant differences (*p*<0.001).

**Table 2:** Cell cycle distribution of SMMC-7721 HCC cells after ICD treatment with gradient concentrations.

Time	Cell cycle	ICD (µg/ml)			
		0	200	300	400
**9 h**	**G1**	25.231±5.057	15.451±0.118	11.864±1.171	16.291±0.012
	**S**	12.134±0.262	12.889±1.016	16.750±2.054	11.656±0.224
	**G2/M**	62.635±4.974	**71.66±0.898** *	**71.386±2.936** *	**72.052±0.235** *
**12 h**	**G1**	83.348±0.645	28.340±2.718	13.447±1.667	4.669±0.720
	**S**	5.021±0.891	10.946±0.572	12.219±1.749	27.294±0.954
	**G2/M**	11.629±1.453	**61.102±3.441** **	**74.332±0.506** **	**68.036±0.308** **
**15 h**	**G1**	88.429±1.526	59.165±1.263	15.836±0.396	10.784±0.114
	**S**	5.879±0.708	4.383±1.651	11.005±2.153	10.365±1.636
	**G2/M**	5.681±1.734	**36.450±0.486** **	**73.158±2.024** **	**78.851±1.522** **
**18 h**	**G1**	89.390±2.546	78.298±0.123	17.418±0.554	11.124±0.096
	**S**	5.899±2.133	2.263±0.370	14.194±0.882	12.381±0.897
	**G2/M**	4.709±1.823	**19.438±0.480** **	**68.388±0.327** **	**76.494±0.800** **

Data are mean ± SD of three independent experiments.

* Significant differences (*p*<0.05),

** Significant differences (*p*<0.001).

### ICD Inactivates Cdc25C by Activating the Chk1/Chk2/Cdc25C Pathway

**Figure 4:**
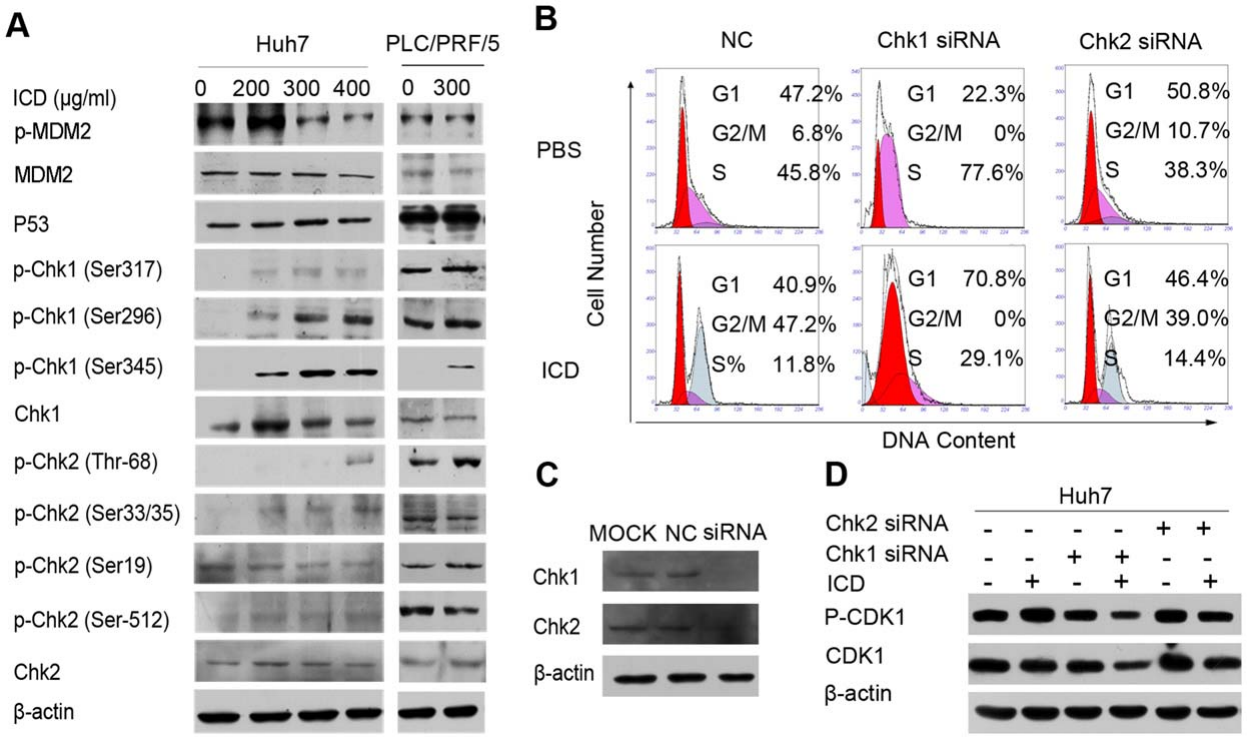
The relative contributions of the Chk1, Chk2 and p53 pathways. (A) Western blot analysis of the activation of p53, MDM2, Chk1 and Chk2 in Huh7 and PLC/PFR/5 cells after ICD treatment for 48 h. (B) The cell cycle distribution of Huh7 cells after ICD treatment for 18 h following siRNA transfection. (C) Western blot analysis of the silencing effect of Chk1 or Chk2 expression levels after transfection with their corresponding siRNA. (D) Western blot analysis of p-CDK1 or CDK1 expression levels after ICD treatment for 18 h following transfection with their corresponding siRNA.

It is well known that CDK1/cyclin B1 complex can be inactivated by the Chk1/Chk2/Cdc25C or p53 pathways. Therefore, to elucidate which pathway is involved in the phosphorylation of CDK1 observed in our experiments, we determined the levels of the phosphorylated forms of these molecules by Western blotting. Our results indicate that ICD does not alter the protein level of p53 (either wild type or mutant), although the p-MDM2 expression level was decreased. In ICD-treated Huh7 cells, we observed a dose-dependent upregulation of the phosphorylation of Chk1 (at Ser317, Ser345 and Ser296). ICD also increased the phosphorylation of Chk2 at Thr68, but it had no effect on other phosphorylation sites within this protein. In PLC/PFR/5 cells, we also observed the upregulation of p-Chk1 (at Ser345) and p-Chk2 (Thr68) after ICD treatment (Figure 4A). These results suggest that the inactivation of CDK1 observed with ICD treatment is mainly induced by Chk1- and Chk2-mediated phosphorylation.

Because both Chk1 and Chk2 were phosphorylated after ICD treatment, to further determine the relative contribution of Chk1 and Chk2 to ICD-induced G2/M arrest, Huh7 cells were treated with ICD after transfection with either Chk1/Chk2 siRNA or a negative control. The results showed that in the Chk1 and Chk2 knockdown Huh7 cells, the G2/M percentage of negative control (NC), Chk1 siRNA and Chk2 siRNA was 47.2%, 0% and 39.0%, respectively, after ICD treatment. Western blot analysis showed that the expression of p-CDK1 was decreased in the Chk1 knockdown cells compared with the Chk2 or NC after ICD treatment. The results suggest that Chk1 siRNA disrupts the G2/M cell cycle arrest, while the negative control or Chk2 do not.

### ICD Decreases the CD133^+^ Fraction and Tumorigenicity of PLC/PRF/5 CD133^+^ Cells

**Figure 5:**
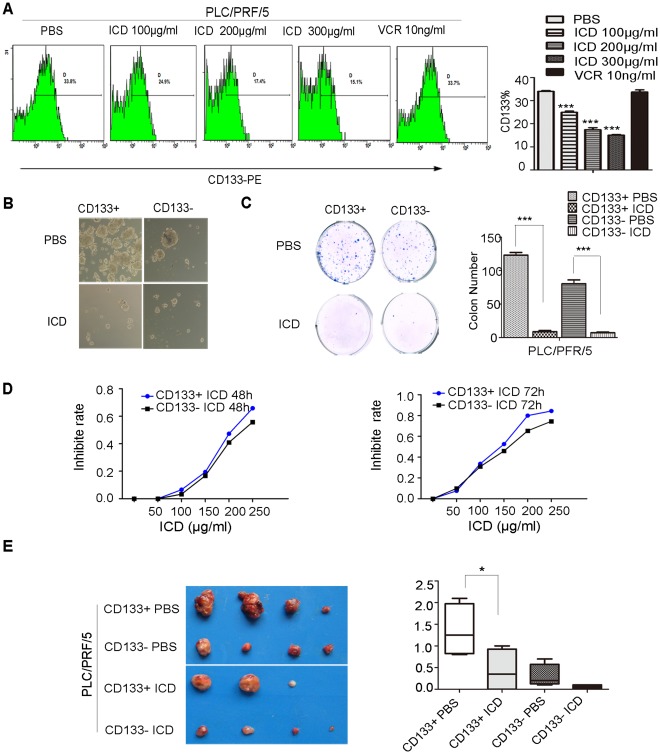
The effect of growth suppression on CD133^+/−^ PLC/PRF/5. (A) PLC/PRF/5 cells were treated with various doses of ICD or 10 ng/ml vincristine for 48 h. The percentage of CD133^+^ cells was then determined by flow cytometry. Statistically significant differences were determined using the Student’s *t*-test (* = *p*<0.05; each point represents the mean ± SD). (B) The hepatosphere formation of PLC/PFR/5 CD133^+/−^ cells that were sorted by FACS and treated with PBS or 150 µg/ml of ICD for 48 h. (C) The colony formation of PLC/PRF/5 CD133^+/−^ cells that were sorted by FACS and then treated with PBS or 150 g/ml ICD for 48 h. (D) The growth inhibition rates of PLC/PFR/5 CD133^+/−^ cells resulting from 48 and 72 h ICD treatments. (E and F) An amount of 5000 PLC/PFR/5 CD133^+/−^ cells/mouse were subcutaneously injected into nude mice. After 2 weeks, 0.4 mg/ml of ICD or PBS was administered 5 times per week for 4 weeks. The resulting tumors were excised from the animals after treatment. Statistical significance was determined using the Student’s *t*-test. Each point represents the mean ± SD.

Based on our findings that ICD can inhibit the proliferation of HCC cell lines *in vitro* and *in vivo*, we investigated whether cancer stem cells (CSCs) were also susceptible. Because CD133 has been shown to be a cancer stem cell marker in HCC, we examined the percentage of CD133^+^ PLC/PRF/5 cells by flow cytometry after a 48 h ICD treatment. We found that the subpopulation of CD133^+^ cells was significantly reduced from 33.8% to 24.9%, 17.4% and 15.1% after treatment with 100, 200 and 300 µg/ml of ICD, respectively (*p*<0.05). In addition, we compared the effect of ICD on the CD133^+^ subpopulation with the conventional chemotherapeutic drug vincristine after treatment for 48 h with a 10 ng/ml dosage. The results showed that vincristine did not affect the percentage of CD133^+^ cells (*p*>0.05) (Figure 5A). ICD also decreased the percentage of EpCAM^+^ cells (EpCAM is another well-known CSC marker in HCC) (Figure S4).

One of the properties of CSCs is their ability to survive under anchorage-independent growth conditions. Therefore, the effect of ICD on hepatosphere formation was determined by the treatment of PLC/PRF/5 CD133^+/−^ subpopulations with ICD or PBS for 48 h. Compared with the PBS control, we found that 150 µg/ml of ICD remarkably inhibited hepatosphere formation and colony formation in cells derived from the CD133^+^ and CD133^−^ subpopulations (Figure 5B, C).

CD133^+^ cells are more resistant to routine anti-cancer drugs compared to CD133^−^ fraction. Therefore, the growth inhibitory effects of CD133^+^ and CD133^−^ cells induced from ICD treatment was determined by MTT assay. CD133^+^ PLC/PRF/5 cells were more sensitive to ICD than the CD133^−^ cells with time (Figure 5D).

It is unknown whether ICD can suppress the tumorigenicity of CD133^+^ HCC cells. Therefore, PLC/PRF/5 CD133^+/−^ cells were injected into NOD/SCID mice. Two weeks post-injection, ICD was administered peritoneally at a dosage of 0.4 mg/mouse 5 times a week for 4 weeks. The results demonstrated that ICD remarkably decreased the tumorigenicity of CD133^+^ PLC/PRF/5 cells (Figure 5E).

## Discussion

DLF (*Dicranostigma leptopodum (Maxim.) Fedde* **)** has been used in the clinical treatment of pulmonary tuberculosis and has been shown to induce apoptosis in SMMC-7721 human hepatoma cells [11], an effect that was also observed in the present study; however, Zhang *et al.* only determined the effects of the entire DLF extracts mixture on one human HCC cell line and did not examine the effects of any DLF alkaloid monomers. Therefore, to our knowledge, the present study is the first to elucidate the anti-cancer effects of an alkaloid monomer, ICD, in DLF extracts.

Of the five main components in DLF extracts, only ICD treatment resulted in an obvious inhibition of proliferation at a relatively low concentration. Therefore, we focused on the mechanism behind the anti-tumoral properties of ICD. It has been reported that ICD can affect the contraction of rabbit oviduct smooth muscle [12] as well as the action potentials of isolated canine Purkinje fibers and ventricular muscles [13]. ICD treatment also imparted relaxant properties on the rat aorta [14]. Additionally, ICD can effectively bind to DNA, thus behaving as a typical intercalating agent [15].

ICD can inhibit the proliferation of HCC cell lines with an IC_50_ of 200∼300 µg/ml. By contrast, L-02 cells were found to be remarkably resistant to this compound. In L-02 cells, the observed inhibitory rate was less than 1% at such doses, indicating that ICD may be less toxic to normal cells than to cancer cells. Therefore, ICD may not exhibit toxicity in experimental animals. The AKT pathway has profound effects on cell proliferation, and the inhibition of this pathway is beneficial in the treatment of cancer [16], [17]. In the present study, ICD treatment did not alter the expression or the phosphorylation levels of AKT, S6 or Erk1/2, suggesting that the observed inhibition of proliferation may not occur via these pathways.

Many anti-cancer drugs cause cell death through the induction of apoptosis [18]. Early in the apoptotic process, phosphatidylserine (PS) becomes exposed on the cell surface. This event is thought to be important for the ability of macrophages to recognize apoptotic cells. PARP, which helps to maintain cell viability, is one of the main cleavage targets of caspase-3. The cleavage of PARP facilitates cellular disassembly and is a useful marker for cell apoptosis [19], [20]. The apoptosis evoked by ICD was confirmed by the Annexin V/7-AAD double-staining method as well as through the examination of cleaved PARP levels. Here, ICD increased the number of Annexin V-positive cells and led to an upregulation of cleaved PARP levels. Because ICD suppressed cell growth and induced apoptosis in HCC cell lines, we hypothesized that it might inhibit HCC tumorigenicity *in vivo*. In our mouse xenograft study, ICD significantly reduced the tumor volume without significantly affecting body weight.

The G2/M checkpoint allows the cell to repair DNA damage before entering mitosis. The stepwise activation of CDK1 is essential for cells to correctly enter the M phase [21]. This process involves the formation of a complex between CDK1 and cyclin B1 or cyclin A. CDK1 is subsequently activated via a Cdk-activating enzyme, which phosphorylates the activating residues on CDK1. Inhibitory phosphorylation can also be performed at Thr160/161, Thr14 and Tyr15 by Wee1 [5] and Myt1 [6]. The phosphatase Cdc25C, by contrast, can dephosphorylate Thr14 and Tyr15 [22]. Cdc25C itself can be inactivated by the Chk1 kinase, which phosphorylates Ser216 on Cdc25C. Chk2 subsequently binds to members of the 14-3-3 protein family, resulting in the sequestration of Cdc25C in the cytoplasm and the prevention of premature mitosis. The activation of CDK1/cyclin B1 can also be prevented by p53 [23]. According to our results, ICD treatment induced a pronounced G2/M phase arrest by increasing the level of cyclin B1 as well as the accumulation of Thr14/Tyr15-phosphorylated CDK1. The expression level of cyclin A was not influenced by ICD treatment. The increased level of the inactive CDK1/cyclin B1 complex resulted in the decreased expression of E2F1 and, thus, an inhibition of its function. ICD treatment also led to an upregulation of Wee1, Myt1 and Ser216-phosphorylated Cdc25C (Ser216) levels. Our studies therefore suggest that ICD inactivates the CDK1/cyclin B1 complex by inactivating Cdc25C. This results in the downregulation of E2F1 expression and G2/M arrest.

The Chk1/Chk2 proteins participate in the transition between the G2 and M phases and are potential targets for cancer therapy [24], [25]. These kinases are activated upon DNA damage, which results in the inactivation of Cdc25C. Chk1 is activated by phosphorylation at Ser317, Ser345 and Ser296, while Chk2 is activated at Ser33/35, Ser516, Ser296 and Thr68 [26]. In our current study, p-Chk1 (Ser317, Ser345 and Ser296) and p-Chk2 (Thr68) were upregulated after ICD treatment. To determine the relative contributions of these proteins on the ICD-induced cell cycle arrest, we analyzed the cell cycle distribution of siRNA-mediated Chk1 or Chk2 knockdown Huh7 cells after treatment with ICD or PBS. The results showed that Chk1 knockdown disrupts the G2/M arrest compared with NC or Chk2. These results indicate that ICD treatment activates Chk1 and Chk2, allowing these kinases to inactivate Cdc25C. Cdc25C, Wee1 and Myt1 then decrease the activity of the CDK1/cyclin B1 complex, resulting in an arrest of the cell cycle at the G2/M phase.

Although many drugs that block cell cycle processes can inhibit cancer cell growth effectively, they may be more efficient when combined with clinical chemotherapeutic drugs. Stern et al. have reported that methionine-depletion induced S/G2 cell cycle blockage in cancer cells increased the sensitivity to doxorubicin and vincristine [27]. Therefore, ICD may also increase the efficiency of routine drugs, although further study is required to prove this. According to our results, ICD induces the G2/M phase arrest by activating the Chk1/Cdc25C/CDK1 pathway. However, methionine depletion-induced S/G2 blockage occurs via a different mechanism than that of ICD. Due to the central role of DNA methylation to carcinogenesis, cancer cells are more dependent on methionine than normal cells and the growth arrest effect by disrupting methionine metabolism is reversible [28], [29]. This suggests that due to their different mechanisms of blocking the cell cycle, there may be synergistic effects when combining ICD with methionine-depletion to treat tumors.

Cancer stem cells are a small population of cells within a tumor that are thought to drive tumor growth and recurrence [30]. They are capable of self-renewal and show chemoresistance to many current cancer chemotherapeutic drugs [31]. CSCs, which cannot be eliminated by conventional medications, survive to regenerate new tumors [32]. Recently, HCC CSCs have been identified by several markers, such as CD133 [33] and epithelial cell adhesion molecule (EpCAM) [34].

In our present study, we showed that ICD can significantly decrease the percentage of CD133^+^ cells, while vincristine was unable to do so. We also used salnomycin, which was reported to selectively kill CSCs in breast cancer [35], as another positive control. We found that salnomycin decreased the percentage of CD133^+^ cells, but to a lesser extent than ICD at the same concentration. In addition, ICD can also decrease the percentage of cells expressing EpCAM, which is another CSC marker. CSCs are unique in their ability to form hepatospheres under anchorage-independent growth conditions as well as to form colonies [36]. We found that ICD could decrease the ability of PLC/PRF/5 CD133^+^ cells to form hepatospheres as well as their ability to form colonies. CD133^+^ cells are more resistant to conventional drugs such as doxorubicin [31]. In our study, ICD exerted a relatively stronger inhibitory impact on CD133^+^ cells compared with CD133^-^ cells. Furthermore, ICD could remarkably reduce the tumorigenicity of CD133^+^ cells. Further study is required to ascertain the underlying mechanism of the ICD-dependent decrease in the fraction and tumorigenicity of CD133^+^ cells.

In summary, ICD inhibits proliferation in HCC cell lines by inducing a G2/M arrest and subsequent apoptosis. ICD also decreases the tumor volume of xenografts without any adverse effects on body weight in nude mice and NOD/SCID mice. In addition, ICD can decrease the percentage of CD133^+^ cells as well as reduce their ability to form tumor-like spheres *in vitro*. These results indicate that ICD might be therapeutically useful in the treatment of HCC.

## Materials and Methods

### Cell Lines and Cell Culture

The human HCC cell lines SMMC-7721 and Huh7 were provided by the Cell Bank of the Institute of Biochemistry and Cell Biology at the China Academy of Sciences (Shanghai, China). MHCC-97L was obtained from the Liver Cancer Institute of Zhongshan Hospital at Fudan University (Shanghai, China). PLC/PRF/5 and SNU-182 were purchased from the American Type Culture Collection (ATCC) (Manassas, USA). All cell lines were cultured in Dulbecco’s Modified Eagle’s Medium (DMEM) (Sigma-Aldrich, St Louis, MO) containing 10% heat-inactivated fetal bovine serum (FBS) (Hyclone, Logan, UT) and supplemented with 100 IU/ml penicillin G and 100 µg/ml streptomycin (Sigma). All cell lines were incubated at 37°C in a humidified atmosphere with 5% CO_2_.

### Drug Stocks

The *Dicranostigma leptopodum (Maxim) Fedde* (DLF) alkaloid extract was prepared at a stock concentration of 50 mg/ml in PBS and stored in the dark at 4°C. Isocorydine (ICD), isolated from *Dactylicapnos scandens Hutchins* (Shanghai Zhanshu Chemical Sci-Tech Company, China), was diluted at 10 mg/ml in PBS and stored at 4°C in a dark bottle.

### Fluorescence-Activated Cell Sorting (FACS)

The PLC/PFR/5 cells were incubated with a PE-conjugated CD133/1(AC133) antibody (Miltenyi Biotec, Germany) and sorted into CD133^+^ and CD133^−^ cell subpopulations on an Epics Altra flow cytometer (Beckman Coulter, USA). The purity of the sorted cells was evaluated by Western blotting.

### Cell Cycle Analysis

For cell cycle analysis, 2×10^5^ cells were plated in a 6-well culture plate and grown for 24 h. The cells were then incubated with 1 mM thymidine (Sigma-Aldrich) for 24 h to synchronize cells at the G1/S boundary. The cells were then treated with fresh media containing various concentrations of ICD for different times. Next, the cells were trypsinized, washed twice with cold PBS and fixed with cold 70% ethanol at −20°C overnight. The cells were then washed twice with PBS and incubated with 10 mg/ml RNase A, 400 mg/ml propidium iodide and 0.1% Triton X in PBS at room temperature (RT) for 30 m. Cells were subsequently analyzed by flow cytometry.

### Apoptotic Assay

Annexin V and 7-AAD staining was used to visualize apoptotic cells according to the manufacturer’s instructions. Briefly, 2×10^5^ cells were seeded in 6-well plates and treated with ICD at a concentration of 100, 200 or 300 µg/ml for 48 h. Cells were then collected and washed twice with PBS and resuspended in 400 µl of 1×binding buffer. Next, 5 µl of the Annexin V-PE and 7-AAD solution was added, and samples were incubated for 15 m at RT and analyzed by flow cytometry.

### Western Bblotting

Freshly sorted cells were lysed and subjected to sodium dodecyl sulfate polyacrylamide gel electrophoresis (SDS-PAGE) and electroblotting to nitrocellulose or polyvinylidene difluoride (PVDF) membranes. Proteins on the membranes were immunodetected with various specific primary antibodies and HRP-conjugated secondary antibodies (Table S1). Chemiluminescent detection was performed by the Supersignal West Femto Chemiluminescent substrate kit (Thermo scientific, Cat No. 34095). β-actin was used as a loading control.

### Hepatosphere Formation Assay

PLC/PRF/5 CD133^+/−^ cells that had been sorted by FACS were treated with 150 µg/ml of ICD for 48 h and were then dissociated into single cells. Cells were cultured in suspension in ultra-low-adherence multiwell plates (Costar) with serum-free medium. Half of the media volume was changed every other day for a total of 7 days. Hepatospheres were then counted.

### MTT Assay

Cell toxicity and proliferation after ICD treatment were determined using the MTT assay according to the manufacturer’s specifications. Briefly, 5000 cells/well were plated in triplicate in 96-well plates, and the cells were exposed to an ICD concentration gradient for 24, 48 and 72 h. The MTT reagent was prepared at 5 mg/ml in PBS. This MTT stock solution was then added to each well at a 1∶10 dilution. Cells were incubated for 4 h. Afterward, the resulting crystals were dissolved in 100 µl DMSO, and the absorbance at 570 nm was measured using an ELISA plate reader, with background subtraction measurements performed at 630 nm. The inhibition rate was calculated as follows:

Inhibition rate = 1−(A_570_–A_630_) of treated cells/(A_570_–A_630_) of control cells.

### RNA Interference-based Gene Knockdown Experiment

Small-interfering RNA (siRNA) oligos for Chk1, Chk2 and general negative control were synthesized and annealed by GenePharma (Shanghai, China). The fragments were designed to target Chk1 or Chk2 transcripts.

Chk1: 5′-GACUGGGACUUGGUGCAAAtt-3′ (sense).

5′-UUUGCACCAAGUCCCAGUCtt-3′ (antisense).

Chk2: 5′-GUAAGAAAGUAGCCAUAAATT-3′ (sense).

5′-UUUAUGGCUACUUUCUUACTT-3′ (antisense).

### *In vivo* Tumorigenicity

The tumor suppressive effect of ICD on Huh7 and SMMC-7721 xenografts *in vivo* was determined by subcutaneously injecting 1×10^6^ cells into 6- to 8-week-old male nude mice. Two weeks after the cells were injected, 0.4 mg/mouse ICD or PBS control injections were administered intraperitoneally 5 times per week for 4 weeks. To evaluate the effect of ICD on the tumorigenicity of CD133^+/−^ HCC cells, 6- to 8-week-old male NOD/SCID mice, maintained under standard conditions according to institutional guidelines, were subcutaneously inoculated bilaterally with 5000 sorted CD133^+^ or CD133^−^ PLC/PRF/5 cells in 50 µl of a serum-free DMEM/Matrigel mixture (1∶1) (BD Biosciences). Two weeks later, the mice were injected intraperitoneally with ICD at a dose of 0.4 mg/mouse 5 times per week for 4 weeks. The effect of ICD treatment on tumor growth was evaluated by weighing the tumor masses and subsequent statistical analysis.

All of the animals used in this study were manipulated and housed according to protocols approved by the Shanghai Medical Experimental Animal Care Commission.

### Statistical Analysis

All of the data are presented as the mean ± standard deviation (SD). Statistical analyses (two group comparisons) were performed using the Student’s *t*-test. *p*<0.05 was considered to be statistically significant.

## Supporting Information

Figure S1 **The chemical structure of ICD.**
(TIF)

Figure S2 **Western blot analysis of AKT, p-AKT, S6, p-S6, Erk1/2 and p-Erk1/2 in Huh7, SMMC-7721 and PLC/PRF/5 cell lines after treatment with ICD at a concentration of 250, 200 or 250 µg/ml for 48 h.**
(TIF)

Figure S3 **The apoptosis-related genes of Huh7, SMMC-7721 and PLC/PRF/5 cell lines after ICD treatment for 48 h evaluated using real-time PCR.**
(TIF)

Figure S4 **The percentage of EpCAM expression in Huh7 and SNU-182 cells treated with 200, 300 and 400 µg/ml of ICD for 48 h.** Statistically significant differences were determined using a Student’s t- test (* = *p*<0.05, ** = *p*<0.001, each point represents the mean ± SD).
(TIF)

Table S1 **Antibodies used in this study.**
(DOC)
