# Supplementary figures and images for: Isocorydine Inhibits Cell Proliferation in Hepatocellular Carcinoma Cell Lines by Inducing G2/M Cell Cycle Arrest and Apoptosis
Source: PLoS One. 2012 May 18;7(5):e36808. doi: 10.1371/journal.pone.0036808 (PMC3356335; doi:10.1371/journal.pone.0036808)

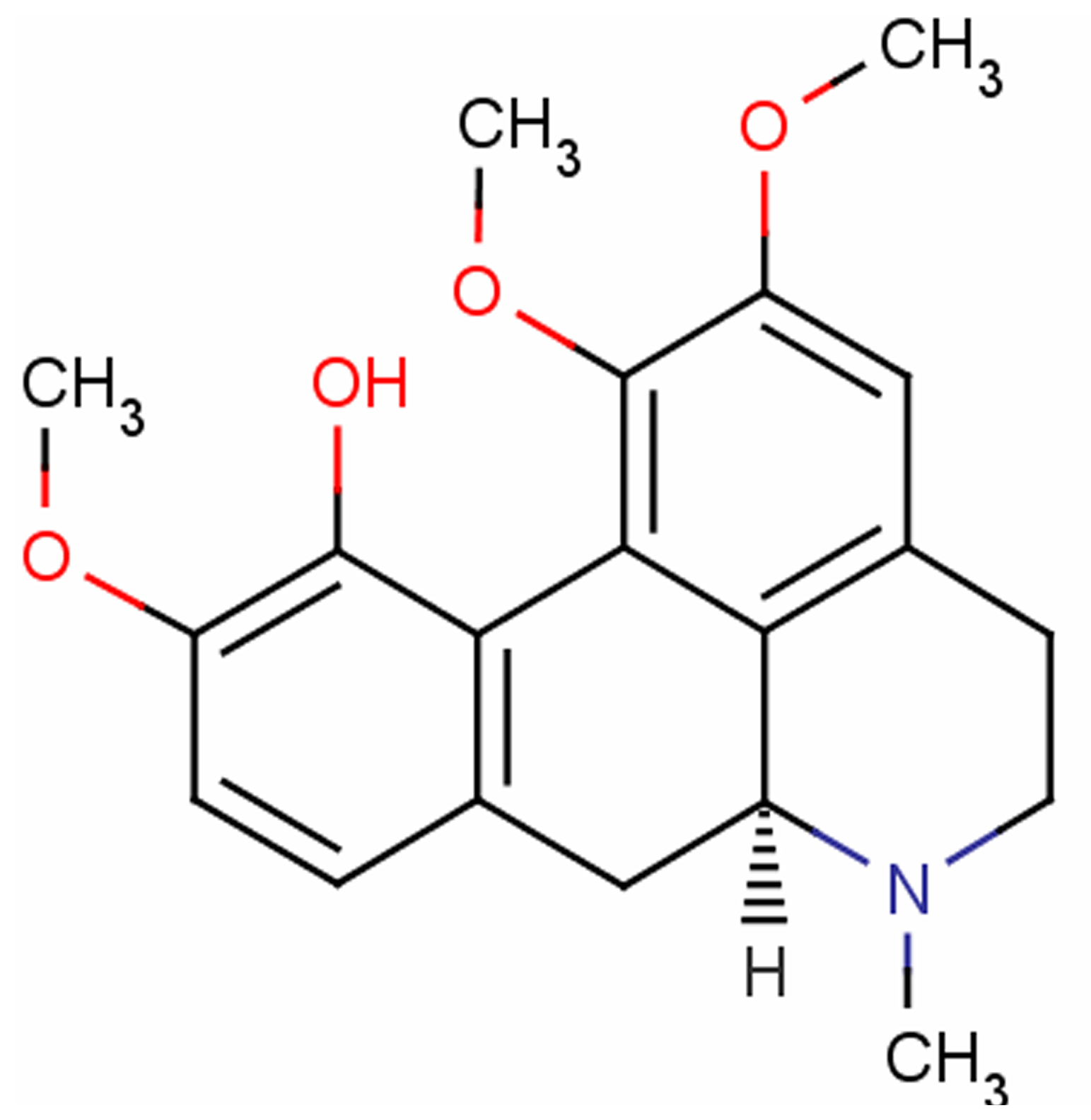

Supplement: Figure S1 — The chemical structure of ICD. (TIF) [file pone.0036808.s001.tif]

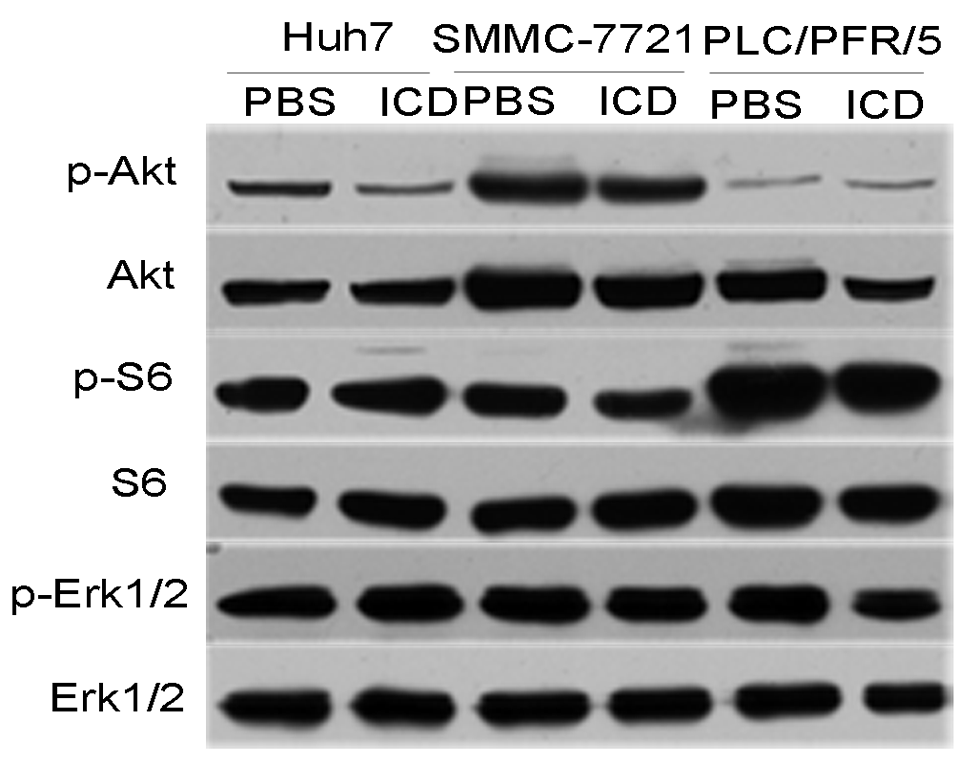

Supplement: Figure S2 — Western blot analysis of AKT, p-AKT, S6, p-S6, Erk1/2 and p-Erk1/2 in Huh7, SMMC-7721 and PLC/PRF/5 cell lines after treatment with ICD at a concentration of 250, 200 or 250 µg/ml for 48 h. (TIF) [file pone.0036808.s002.tif]

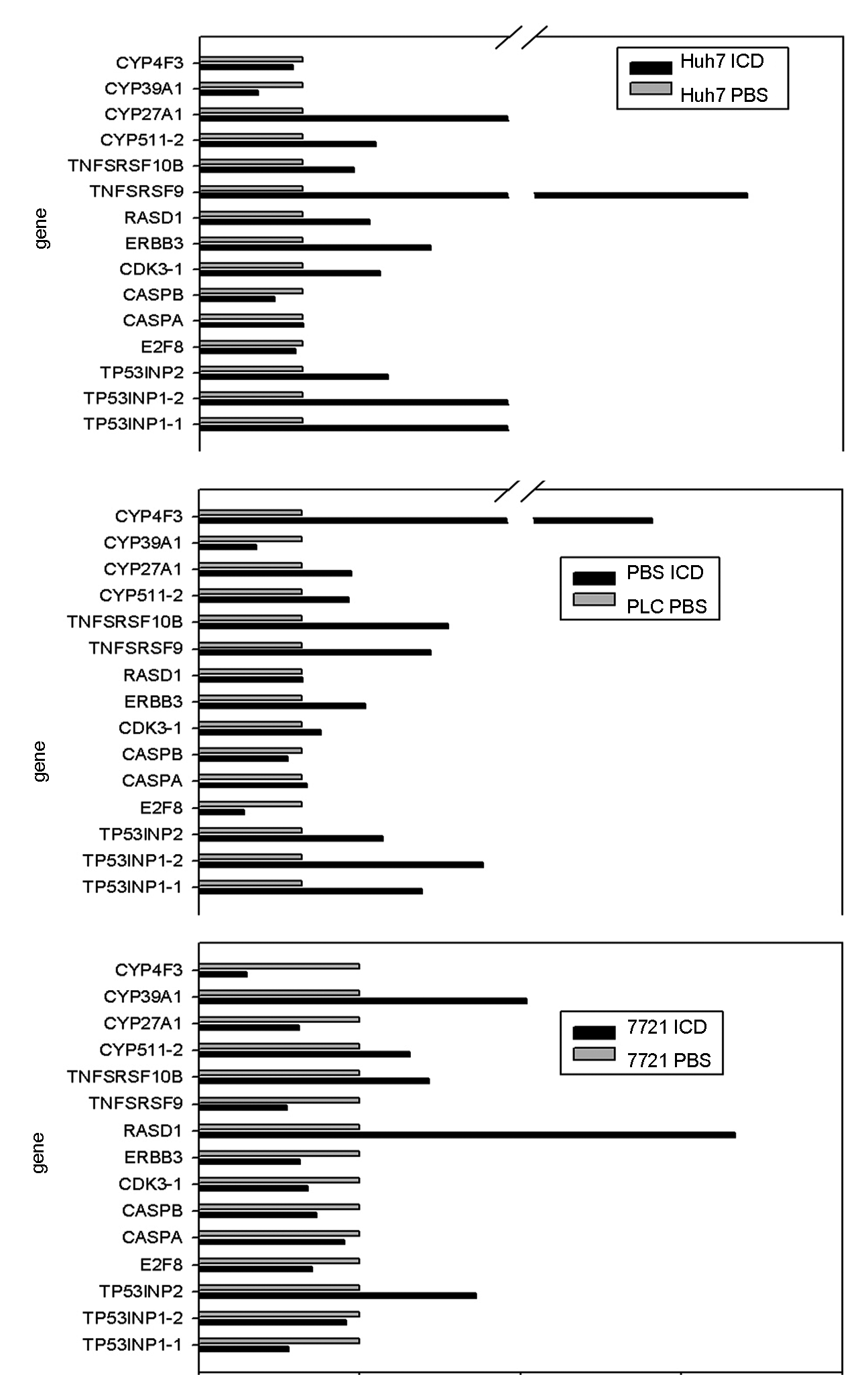

Supplement: Figure S3 — The apoptosis-related genes of Huh7, SMMC-7721 and PLC/PRF/5 cell lines after ICD treatment for 48 h evaluated using real-time PCR. (TIF) [file pone.0036808.s003.tif]

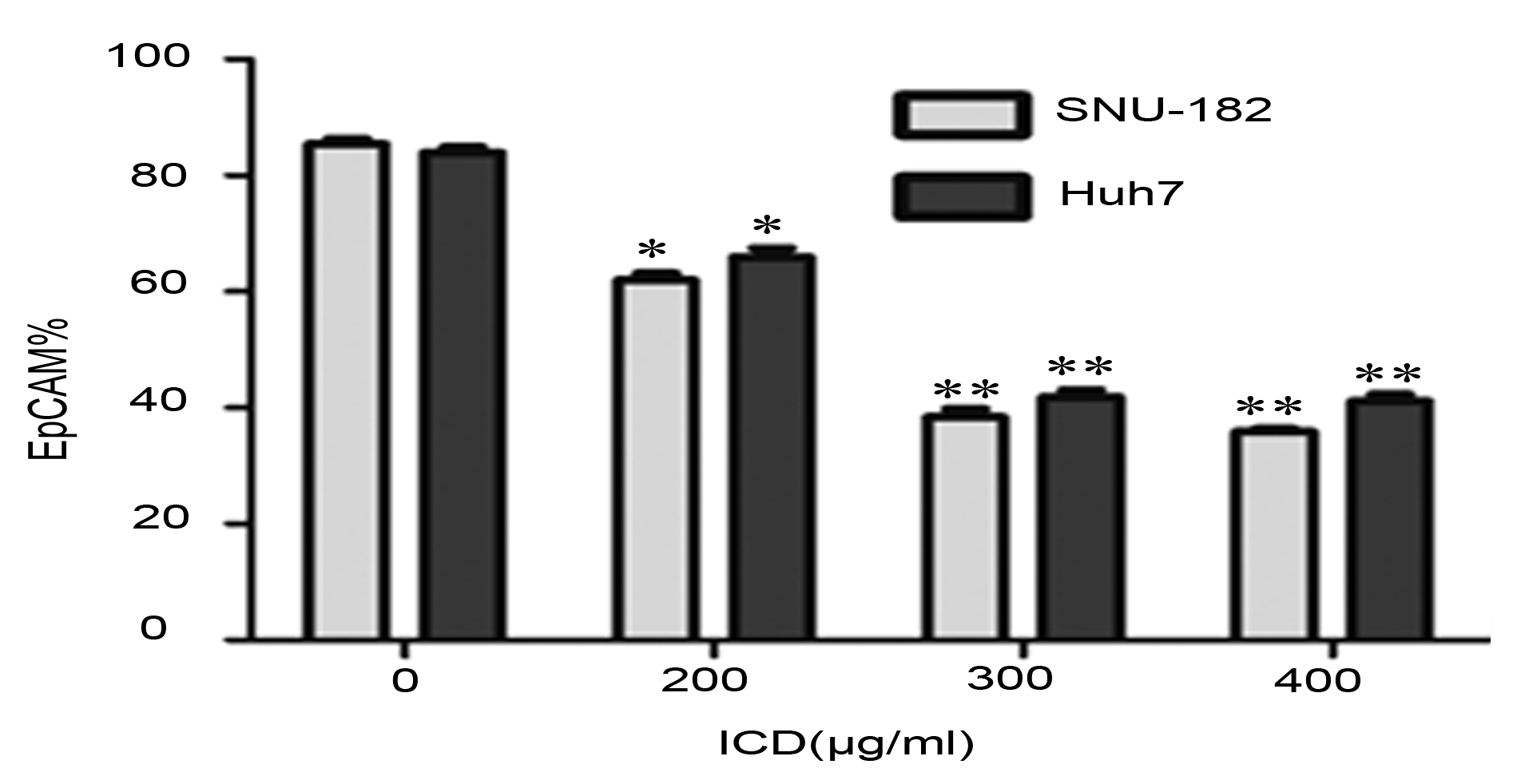

Supplement: Figure S4 — The percentage of EpCAM expression in Huh7 and SNU-182 cells treated with 200, 300 and 400 µg/ml of ICD for 48 h. Statistically significant differences were determined using a Student’s t- test (* = p<0.05, ** = p<0.001, each point represents the mean ± SD). (TIF) [file pone.0036808.s004.tif]
