# Supplementary material for: Isocorydine Inhibits Cell Proliferation in Hepatocellular Carcinoma Cell Lines by Inducing G2/M Cell Cycle Arrest and Apoptosis
Source: PLoS One. 2012 May 18;7(5):e36808. doi: 10.1371/journal.pone.0036808 (PMC3356335; doi:10.1371/journal.pone.0036808)
Supplement: Table S1 — Antibodies used in this study. (DOC) [file pone.0036808.s005.doc]

**Table S1. Antibodies used in this study**

| **Antibody** | **Clone, host** | **Dilution** | **Company** |
| --- | --- | --- | --- |
| p-CDK1 | Tyr-15, rabbit polyclonal | 1:200 for WB | Santa Cruz |
| CDK1 | 17, mouse mAb IgG1 | 1:200 for WB | Santa Cruz |
| Cyclin B1 | GNS1, mouse mAb IgG1 | 1:200 for WB | Santa Cruz |
| Cyclin A | H-432, rabbit polyclonal | 1:200 for WB | Santa Cruz |
| Rb | M-153 rabbit polyclonal | 1:100 for WB | Santa Cruz |
| p-Rb | Ser807/811 rabbit mAb | 1:8000 for WB | Cell Signaling |
| E2F1 | HK95, mouse mAb IgG2a | 1:200 for WB | Santa Cruz |
| Wee1 | B-11, mouse mAb IgG1 | 1:100 for WB | Santa Cruz |
| Cdc25C | 5H9, rabbit polyclonal | 1:500 for WB | Cell Signaling |
| p-cdc25C | 63F9, rabbit polyclonal | 1:500 for WB | Cell Signaling |
| Myt1 | 40A9, rabbit mAb | 1:500 for WB | Cell Signaling |
| p-myt1 | D2E11, rabbit mAb | 1:1000 for WB | Cell Signaling |
| Cleaved PARP | 2D2, mouse mAb IgG1 | 1:200 for WB | Cell Signaling |
| Phosphor-Chk1/Chk2 antibody sample kit | rabbit polyclonal | 1:300 for WB | Cell Signaling |
| AKT | C67E7, rabbit mAb | 1:1000 for WB | Cell Signaling |
| p-AKT | 9271, rabbit polyclonal | 1:1000 for WB | Cell Signaling |
| Erk1/2 | 137F5, rabbit mAb | 1:1000 for WB | Cell Signaling |
| p-Erk1/2 | 20G11, rabbit mAb | 1:1000 for WB | Cell Signaling |
| S6 | 49D7, rabbit mAb | 1:1000 for WB | Cell Signaling |
| p-S6 | D57.2.2E, rabbit mAb | 1:1000 for WB | Cell Signaling |
| β -actin | AC-15, mouse mAb | 1:30000 for WB | Sigma (St. Louis, MO) |

WB: Western blotting
